# Exploring the relationship between auditory hallucinations, trauma and dissociation

**DOI:** 10.1192/bjo.2020.31

**Published:** 2020-05-20

**Authors:** Deborah Wearne, Guy J. Curtis, Peter Melvill-Smith, Kenneth G. Orr, Annette Mackereth, Leon Rajanthiran, Sean Hood, Winston Choy, Flavie Waters

**Affiliations:** School of Medicine, University of Western Australia, Perth, Australia; School of Psychological Science, University of Western Australia, Perth, Australia; School of Medicine, University of Western Australia, Perth, Australia; Marian Center, Perth, Australia; Marian Center, Perth, Australia; Marian Center, Perth, Australia; Faculty of Health and Medical Sciences, University of Western Australia, Perth, Australia; School of Medicine, University of Western Australia, Perth, Australia; School of Psychological Sciences, University of Western Australia, Perth; and Clinical Research Centre, Graylands Campus, North Metropolitan Health Service, Mental Health, Perth, Australia.

**Keywords:** Post-traumatic stress disorder, trauma, dissociative disorders, childhood experience, comorbidity

## Abstract

**Background:**

It is clinically imperative to better understand the relationship between trauma, auditory hallucinations and dissociation. The personal narrative of trauma has enormous significance for each individual and is also important for the clinician, who must use this information to decide on a diagnosis and treatment approach.

**Aims:**

To better understand whether dissociation contributes in a significant way to hallucinations in individuals with and without trauma histories.

**Method:**

Three groups of participants with auditory hallucinations were recruited, with diagnoses of: schizophrenia (without trauma) (*n* = 18), post-traumatic stress disorder (PTSD, *n* = 27) and comorbid schizophrenia and PTSD (SCZ+PTSD), *n* = 26). Clinician-administered measures included the PTSD Symptoms Scale Interview (PSSI-5), the Clinician-Administered Dissociative States Scale (CADSS) and the Psychotic Symptom Rating Scales (PSYRATS).

**Results:**

Dissociative symptoms were significantly higher in participants with trauma histories (PTSD and SCZ+PTSD groups) and significantly correlated with hallucinations in trauma-exposed participants, but not in participants with schizophrenia (without trauma history). Hallucination severity was correlated with the CADSS amnesia subscale score, but depersonalisation and derealisation were not.

**Conclusions:**

Dissociation may be a mechanism in trauma-exposed individuals who hear voices, but it does not explain all hallucinatory experiences. The SCZ+PTSD group were in an intermediary position between schizophrenia and PTSD on dissociative and hallucination measures. The PTSD and SCZ+PTSD groups experienced dissociative phenomena much more frequently than the schizophrenia group, with a significant trend towards the amnesia subtype of dissociation**.**

Auditory hallucinations and the experience of trauma attend individuals who suffer a broad variety of psychiatric disorders, including schizophrenia and post-traumatic stress disorder (PTSD).^1^ An association between hallucinations and trauma has been documented in PTSD, where two-thirds of civilian trauma survivors and half of traumatised military veterans report hearing voices.^2^ Such findings have led to suggestions that trauma might be a direct cause of auditory hallucinations.^3,4^ Because dissociation is a common response to trauma, one hypothesis is that trauma causes hallucinations through the process of dissociation, which either impairs the integration of traumatic experience with components of the self identity^4,5^ or causes a failure of contextual integration of present and past experiences, resulting in involuntary intrusions.^6^ In support, robust associations between voice hearing and dissociation (*r* = 0.52) have been confirmed in a meta-analysis involving 31 studies,^7^ suggesting that dissociation (and particularly depersonalisation^8^) may be a mediating factor between trauma and certain types of hallucinatory experiences. However, heterogeneity in the results, methodological limitations and a lack of direct evidence in schizophrenia have led to calls for more research in this area. Furthermore, recent research has questioned whether trauma is necessarily involved in all hallucinations, or whether instead there may be different causal pathways for different types of hallucination.^3^

The present study sought to broaden the understanding of the relationship between dissociation, auditory hallucinations and trauma, using carefully selected participant samples and validated measures. The experience of hallucinations and dissociative state symptoms were examined in three groups of participants with auditory hallucinations and with different diagnoses and trauma histories: schizophrenia (without trauma/PTSD), PTSD with dissociation, and comorbid schizophrenia and PTSD (SCZ+PTSD). Levels of dissociation and hallucination scores were compared between groups, and the strength of the relationship between dissociation and hallucinations was assessed using correlation analyses. Subtypes of symptoms of dissociation were examined using regression analysis. This information can be used to inform clinical management of complex cases involving hearing voices, dissociation and trauma.

## Method

### Participants

Participants (*n* = 71) were recruited from psychiatric services in Perth, Western Australia. Clinical diagnosis was established according to DSM-5^9^ criteria by their treating psychiatrist. Inclusion criteria included the presence of auditory hallucinations, a diagnosis of schizophrenia and/or PTSD with dissociation, age >18 years and capacity to give informed consent. Exclusion criteria included involuntary status or comorbid substance misuse.

### Measures

All validated measures were completed with all participants in one session by the treating clinician, and in the following order:

1. PTSD Symptoms Scale Interview (PSSI-5):^10^ this is a validated semi-structured 17-item scale which assesses PTSD diagnosis and severity on the basis of DSM-5 criteria. All participants were assessed for lifetime traumatic events where trauma was experienced, witnessed or confronted and accompanied by an intense fear, helplessness or horror. The absence/presence of trauma experience as assessed by the PSSI-5 was used to assign participants with schizophrenia to diagnostic subgroups. Participants presenting with complex and multiple traumas were asked to focus on their worst traumatic experience for the assessment.
2. Psychotic Symptom Rating Scales (PSYRATS), hallucinations subscale:^11^ this instrument comprises 11 items designed to quantify multiple dimensions regarding hallucinations, including frequency, duration, location, loudness, belief regarding origin, negative content, controllability, distress and disruption to life.
3. Clinician-Administered Dissociative States Scale (CADSS), subjective items:^12^ this instrument measures present state dissociative symptoms with 23 items under clinician guidance. Subjective items were used to explore the phenomenology of the experience. Participants were given explicit instructions to focus their attention on their experience of hearing voices while completing this measure. This scale immediately followed the PSYRATS to encourage this focus of attention. Subscales assessed were amnesia (2 items, 14 and 15), derealisation (12 items, 1, 2, 8–13, 16–19) and depersonalisation (5 items, 3–7);^12^ items 20–23 were not included in the subscale analysis.
4. Positive and Negative Syndrome Scale (PANSS):^13^ This widely used scale measures positive and negative psychosis symptom severity and general psychopathology with 28 items on a 7-point scale.

### Ethics approval

Signed informed consent was obtained from all participants. Ethics approval was obtained from St John of God Hospital (ID 1459) and Joondalup Health Campus Committee (ID 1834) research ethics committees and the National Health and Medical Research Council (NHMRC) (DW01847).

### Statistical analyses

Demographic and clinical variables and total scores were analysed using analysis of variance (ANOVA) for continuous variables and chi-squared for categorical variables. The association between hallucinations and dissociation was examined using group comparisons, correlations and regressions. Data were sufficiently normally distributed to satisfy statistical assumptions for parametric tests and Pearson's product correlations were conducted. To explore which dissociation subtypes affected hallucination severity, regressions were conducted. Using Gignac & Szodorai's^14^ determination that correlations ≥0.3 can reasonably be considered large, we examined the number of CADSS items that correlated ≥0.3 with PSYRATS scores. We then conducted stepwise regressions of the PSYRATS scores on CADSS items that correlated ≥0.3. Finally, to examine whether dissociation (CADSS) mediated the relationship between trauma (PSSI-5) and voice hearing (PSYRATS) for the PTSD and SCZ+PTSD participants, mediation analysis was run using PROCESS model 4 by Hayes^15^ in SPSS with the three CADSS subscales as potential mediators and 5000 bootstrapped resamples.

## Results

On the basis of the treating psychiatrists’ clinical knowledge and instrument scores, three diagnostic groups of participants were identified: schizophrenia without trauma/PTSD (*n* = 18), PTSD with dissociation but no psychosis (*n* = 27) and comorbid schizophrenia and PTSD (SCZ+PTSD, *n* = 26). Table 1 shows that there were non-significant group differences in age and gender between the three groups and that the two PTSD groups did not differ significantly in mean age at which trauma was experienced.

**Table 1:** Demographic and questionnaire score^a^ comparisons among the three participant groups

	PTSD (*n* = 27), mean (s.d.)	Schizophrenia without trauma history (*n* = 18), mean (s.d.)	Comorbid schizophrenia and PTSD (*n* = 26), mean (s.d.)	*F*(2,68)	*P*
Age, years	42.39 (13.44)	40.81 (15.44)	46.59 (13.67)	1.15	0.324
Age at which trauma experienced, years^b^	14.81 (15.36)	NA	12.58 (10.63)	0.37	0.54
PSYRATS score	39.15_b_ (12.84)	46.78_a_ (6.05)	43.42 (9.46)	3.10	0.051
PSSI-5 score^b^	70.15_a_ (12.50)	0	54.96_b_ (17.59)	13.36	0.001
PANSS total score	45.92_b_ (13.12)	55.84_a_ (9.92)	62.08_a_ (13.76)	26.52	<0.001
PANSS-P score	12.50_b_ (4.70)	19.35_a_ (4.55)	20.85_a_ (4.00)	4.78	0.011
PANSS-N score	22.84_b_ (8.72)	30.64_a_ (6.32)	30.64_a_ (10.72)	20.77	<0.001
PANSS-G score	7.72_b_ (4.20)	15.48_a_ (4.00)	14.64_a_ (5.40)	11.34	<0.001
CADSS total score	42.62_a_ (15.99)	11.94_c_ (7.81)	31.48_b_ (16.87)	23.56	<0.001
CADSS amnesia score	3.30_a_ (2.96)	1.55_b_ (1.94)	3.53_a_ (3.16)	2.96	0.059
CADSS depersonalisation score	12.41_a_ (5.00)	2.67_c_ (2.28)	7.58_b_ (5.02)	25.81	<0.001
CADSS derealisation score	17.11_a_ (8.51)	5.47_b_ (3.52)	13.40_a_ (7.83)	14.24	<0.001
				*χ^2^*(2,71)	*P*
Male, *n*	6	8	10	2.78	0.249
Female, *n*	21	10	16		

CADSS, Clinician-Administered Dissociative States Scale; PANSS, Positive and Negative Syndrome Scale; PANSS-P, positive symptoms, PANSS-N, negative symptoms; PANSS-G, general psychopathology; PSSI-5, 5-item PTSD Symptoms Scale Interview; PSYRATS, Psychotic Symptom Rating Scales; PTSD, post-traumatic stress disorder.

a. Subscript a > b > c indicate that means in same row are significantly different *P* < 0.05.

b. *F*-ratio d.f. = 1.51.

*P < 0.05 based on Tukey's least significant difference (LSD) *post hoc* test.

### Trauma scores and type (PSSI-5)

Both the PTSD and SCZ+PTSD groups had severe PTSD (average scores >46), although PSSI-5 scores were significantly higher in the PTSD than in the SCZ+PTSD group (Table 1). The schizophrenia group scored 0, denoting an absence of trauma history. The type of worst trauma experience was similar between the PTSD and SCZ+PTSD groups. In the PTSD group, 70% of the trauma was experienced in childhood and 84% of the trauma was sexual abuse. The 30% of adult trauma was represented by adult rape, witness of accidental death and refugee war trauma. In the SCZ+PTSD group, 66% of the trauma was childhood abuse, of which 76% was sexual abuse. The 34% of adult trauma included witness of accidental death, experience of sexual or physical abuse and refugee war trauma.

### Positive and negative symptoms (PANSS)

The SCZ+PTSD and schizophrenia groups had significantly greater clinical severity of symptoms on the PANSS than the PTSD group.

### Hallucination severity (PSYRATS)

The PSYRATS scores were similar between groups, although total scores tended to be higher in the schizophrenia and SCZ+PTSD groups compared with the PTSD group, the differences just reaching statistical significance (*P* < 0.05).

### Dissociation (CADSS)

Total dissociation scores were significantly higher for the PTSD group than the SCZ+PTSD group, which in turn were significantly higher than for the schizophrenia group. On the CADSS depersonalisation subscale, the PTSD group scored significantly higher than the SCZ+PTSD group, but the amnesia and derealisation subscale scores were equivalent in the PTSD and SCZ+PTSD groups (Table 1).

### Correlations: hallucination severity (PSYRATS) and trauma severity (PSSI-5)

The PSYRATS and PSSI-5 scores were positively correlated in the PTSD (*r* = 0.55, *P* < 0.001) and SCZ+PTSD (*r* = 0.82, *P* < 0.001) groups, such that higher trauma scores were clearly associated with increased severity of hallucinations.

### Correlations: hallucination severity (PSYRATS) and dissociation subscales (CADSS)

Total CADSS scores correlated significantly with PSYRATS scores in the PTSD and SCZ+PTSD groups, but not in the schizophrenia group (Table 2). In the PTSD and SCZ+PTSD groups, there were significant correlations between the PSYRATS scores and the CADSS subscale of amnesia, but the correlation between PSYRATS scores and CADSS subscales of depersonalisation and derealisation did not reach significance. There were no significant correlations in the schizophrenia group using both total and subscale CADSS scores.

**Table 2:** Correlations between variables by the three participant groups

Correlated variables	PTSD (*n* = 27)	Schizophrenia (*n* = 18)	Comorbid schizophrenia and PTSD (*n* = 26)
PSYRATS–CADSS-total scores	**0.54**^a^	0.29	**0.37**
PSYRATS–CADSS-amnesia scores	**0.44**	0.10	**0.55**
PSYRATS–CADSS-depersonalisation scores	0.36	−0.03	0.37
PSYRAT–CADSS-derealisation scores	0.37	0.42	0.24

CADSS, Clinician-Administered Dissociative States Scale; PSYRATS, Psychotic Symptom Rating Scales; PTSD, post-traumatic stress disorder.

a. Bold denotes significant correlations, *P* < 0.05.

### Regression: dissociation subscale (CADSS) and hallucination severity (PSYRATS) scores

CADSS items were entered in order of correlation strength into three stepwise regressions (one per diagnostic category). Only three CADSS items correlated ≥0.3 with PSYRATS scores for participants in the schizophrenia group, whereas nine and seven CADSS items respectively met this criterion for the PTSD and SCZ+PTSD groups. Table 2 shows significant correlations between the PSYRATS and the CADSS amnesia subscale in the PTSD and SCZ+PTSD groups. In the PTSD participants, CADSS item 14 (*β* = 0.44) (Have there been things which have happened during this assessment that now you can't account for?) and item 21 (*β* = 0.53) (Do you feel confused about who you really are*?*) predicted 48.3% of the variance in PSYRATS scores (*F*[2,24] = 13.14, *P* < 0.001). In the SCZ+PTSD participants, CADSS item 15 (*β* = 0.61) (Have you spaced out, or in some other way lost track of what was going on during this experience?) alone predicted 35% of the variance in PSYRATS scores (*F*[1,24] = 14.46, *P* = 0.001) while no further items significantly added to the prediction of PSYRATS scores in this group.

In the schizophrenia group, there were no significant correlations with any of the CADSS subscale scores (Table 2), although two individual items from the CADSS derealisation and depersonalisation subscales predicted 51.1% of the variance in PSYRATS scores (*F*[2,16] = 9.3, *P* = 0.003): item 1 (*β* = 0.60) (Do things seem to be moving in slow motion?) and item 6 (β = −0.42) (Do you feel disconnected from your own body?).

### Does dissociation mediate the relationship between trauma and voice hearing?

A mediation analysis examined whether dissociation (CADSS) mediated the relationship between trauma (PSSI-5) and voice hearing (PSYRATS) in the PTSD and SCZ+PTSD groups together. The effect of PSSI-5 on PSYRATS was significant (*c* = 0.62, *t*[52] = 3.89, *P* = 0.003), and this was partially mediated by CADSS amnesia (*c*′ = 0.54, *t*[52] = 2.82, *P* = 0.069; *a*_1_*b*_1_ = 0.22 (bootstrapped 95 CI: 0.10–0.42)). CADSS derealisation and depersonalisation were not significant mediators of the relationship between trauma and voice hearing.

## Discussion

This study examined whether dissociation plays a central role in the experience of hallucinations, by examining state dissociation experiences in three groups of participants who hear voices but differ in terms of trauma experience. The levels of dissociation in the PTSD and the SCZ+PTSD groups were significantly higher than in the schizophrenia group (who had no trauma history). The findings also showed a significant correlation between the severity of dissociation and severity of experience of hallucinations in the trauma-exposed groups (PTSD and SCZ+PTSD), but not in the schizophrenia group.

### Trauma, dissociation and hallucinations

The PTSD and SCZ+PTSD groups showed increased dissociation, specifically amnesia, linked to an increase in severity of hallucinations, supporting the mediating role of dissociation in individuals who have been exposed to trauma. There is now evidence that childhood adversity has occurred at significantly higher rates in people with psychosis. Studies have reported a 47% rate of childhood sexual abuse in populations with psychotic illness^16^ and that early trauma exposure significantly increases the risk for psychosis.^17^ However, although trauma can never been ruled out, not all individuals who experience hallucinations report trauma.^3^

In contrast, the subset of participants with schizophrenia without trauma in our study showed a lack of correlation between dissociation and hallucination scores, suggesting that trauma (and the process of dissociation) is not always necessary for auditory hallucinations to occur, as has been previously suggested.^4^ This result provides potential support for the theory that there are multiple pathways to hearing voices.^2^ Outside of the psychosocial stressors and personal narratives of voice hearers, neurobiological mechanisms in schizophrenia clearly link to auditory hallucinations, including frontotemporal connectivity alterations affecting language processing networks and loss of sensory anchors acting to increase dependence on prior expectations.^18^

### Comorbid schizophrenia and PTSD

A substantial proportion of our participants had comorbid schizophrenia and PTSD (SCZ+PTSD). This is consistent with the literature, which suggests that prevalence rates of PTSD in schizophrenia spectrum disorders are fairly high, varying between 0 and 55%, with 78% of papers reporting >10%.^19^ This group had significantly higher scores on the PANSS and its three subscales, in line with studies showing that the severity of trauma and illness factors can result in increased symptom severity and increased comorbidity.^19^ Yet this group had intermediary results in the severity of dissociation and hallucinations. This introduces the interesting concept of a subgroup of participants who experienced trauma-linked dissociative hallucinations, as well as auditory hallucinations that directly link to their psychotic illness. This concept supports the research in the field suggesting that both biological and psychological treatment strategies are clinically useful in schizophrenia.^4^

### CADSS dissociation subscales: amnesia, derealisation and depersonalisation

The results from our analyses of the dissociation subscales of the CADSS were less clear-cut, which was possibly due to limited statistical power, particularly in the schizophrenia group. In the PTSD and SCZ+PTSD groups, however, there was a significant correlation between hearing voices and amnesia subscale scores. Regression analysis suggested that dissociative hallucinations were best predicted by symptoms of amnesia.

There was a non-significant trend with depersonalisation. In the literature, depersonalisation symptoms have been linked to auditory hallucinations^4,5^ in which the voice is seen to represent a component of self-identity which has been dissociated owing to failure of integration of a traumatic experience.^4^ Depersonalisation also plays a central role in the concept of loss of internal anchors described by Allen et al.^20^

Nevertheless, in this study the evidence of the role of amnesia in mediating voice hearing was considerably stronger, particularly given that mediation analysis is a statistical tool that attempts to describe causality.^21^ Steel et al^6^ have developed a contextual integration model to explain the experience of involuntary intrusion in trauma. This model provides the best fit for the results in this study, where trauma fundamentally disrupts the ability to encode and integrate incoming sensory information with co-occurring data stored in the hippocampus. Clinically, this weakened ability to integrate information would present as amnesia and the sensory involuntary intrusions could present as hallucinations. The dissociative amnesia may represent an attempt of the unconscious to forget, and the potential failure of this suppressive defence process invites the experience of hallucinations as involuntary sensory intrusions.

Regression analysis using item scores in the schizophrenia group showed that hallucinations correlated with a sense of detachment from the world (things moving in slow motion) and had an inverse relationship to depersonalisation (disconnected from their own body). Although changes in the sense of body including a loss of ‘bodily-self’ are a common finding in schizophrenia,^22^ in our study this depersonalisation experience was negatively correlated with hearing voices. In contrast, seeing the world as distant or detached, consistent with the derealisation, was positively correlated. This finding is reflected in the clinical situation, where auditory hallucinations in schizophrenia are often described as ‘real’^23^ and can be experienced with a clear sense of the self ‘having a conversation’, whereas the world is seen as distant and detached.

### Dissociative hallucination process model

**Fig. 1:**
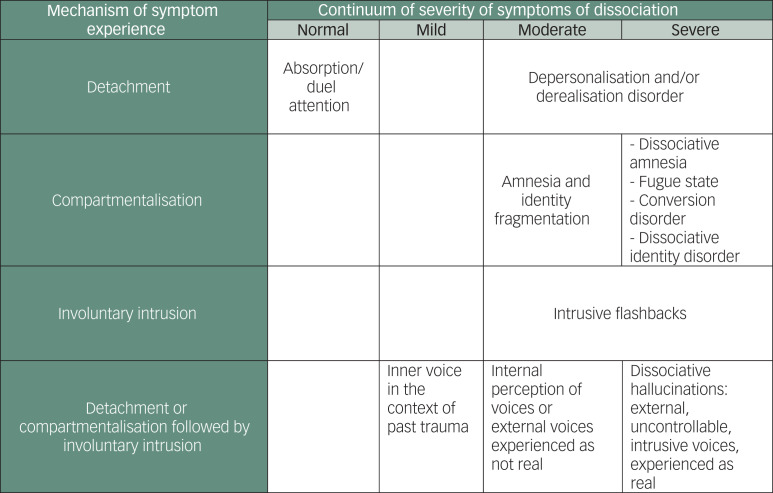
Mechanisms of symptom experience in dissociation. Adapted from Cardena^24^ and Steel et al^6^.

We would suggest that individuals undergo two psychological processes when experiencing dissociative hallucinations (Fig. 1). The first is a loss of integration of consciousness, with either detachment (the subjective experience of an altered mental state) or compartmentalisation (a failure to control that which is normally under voluntary control), as described in Cardena's original model of dissociation.^24^ The second process might involve the involuntary intrusion of sensory, affective or cognitive content into conscious awareness, in order to describe the experience of dissociative hallucinations.^6,25^ These intrusions could be trauma specific, as in dissociative flashbacks, or general, as in dissociative hallucinations. We would see dissociative hallucinations as occurring on a continuum from the experience of internal voices to external, uncontrollable, ego-alien negative voices. Voices may begin with a link to negative self-talk and memories of abuse but can lose that link as anchors fade.

### Limitations

This research has limitations and methodological problems. The subjective experience of dissociation is difficult to operationalise, and despite our efforts to link the symptoms to hearing voices, it is potentially vulnerable to contamination. The use of the DSM-5 definition of trauma in PTSD set specific criteria but potentially leaves out many other trauma experiences. We asked participants specifically about their trauma memories, but recognise that retrospective descriptions of trauma are open to contamination. Individuals with schizophrenia are exposed to trauma, and dissecting comorbidity can be difficult in complex cases. Confounding variables in all participants may have included other diagnoses, such as major depression or generalised anxiety disorder, as only comorbid substance misuse was specifically excluded. It is also important to note that the PSYRATS examines the severity of existing hallucinations, but the process of development of these hallucinations cannot be directly measured.

Sample size was also a significant problem. The small number of participants, especially in the schizophrenia group, affected the statistical power of analyses, particularly when considering correlations among subscales. Small sample size means that sampling variability may affect inferences drawn about the represented population. For example, the participants with PTSD had PSSI-5 scores that represented a severe degree of PTSD symptomology. This increases the likelihood of multiple and complex trauma and means that the data are not necessarily representative of all individuals with PTSD. Group assignment was not random and required clinical accuracy regarding diagnosis, although this problem was mediated by the fact that all the psychiatrists involved had significant (between 10 and 30 years’) clinical experience and were dealing with patients who were clinically known to them.

### Clinical and research implications

If experiences of auditory hallucinations have more than a single causative pathway, it is clinically valid to try to distinguish them. The description of hallucinatory experiences can be similar between individuals who have PTSD and schizophrenia, so it is essential to consider contextual variables such as dissociation symptoms**.** Dissociation is a universal defence mechanism in response to trauma but our groups described differing experiences of it. There is therefore a need to encourage further research into the role of dissociation, particularly amnesia, as a potential mediating factor between hallucinations and trauma.

## Data Availability

Data is available from the authors on request.
